# Merging species? Evidence for hybridization between the eel parasites *Anguillicola crassus* and *A. novaezelandiae* (Nematoda, Anguillicolidea)

**DOI:** 10.1186/1756-3305-5-244

**Published:** 2012-10-30

**Authors:** Daniel S Grabner, Kerstin C Dangel, Bernd Sures

**Affiliations:** 1 University of Duisburg-Essen, Aquatic Ecology, Universitaetsstr. 5, Essen, 45141, Germany

**Keywords:** Nematode, *Anguillicola*, Hybridization, Invasive species, Eel parasite, Lago Bracciano

## Abstract

**Background:**

The eel parasitic nematodes *Anguillicola crassus* (originating from Asia) and *Anguillicola novaezelandiae* (originating from New Zealand) were both introduced to Europe, but occurred in sympatry only in Lake Bracciano in Italy, where they both infected the European eel (*Anguilla anguilla*). *A. novaezelandiae* was introduced to the lake in 1975 and disappeared soon after *A. crassus* was also found there in 1993. We tested the hypothesis if hybridization of the two species might be an explanation for the findings at Lake Bracciano.

**Findings:**

After laboratory infection of one European eel with 10 third stage larvae of each parasite, two living female and 4 male adults of each species were found to co-occur in the swim bladder after 222 days post exposure. In 9 out of 17 eggs, isolated in total from uteri of the two *A. novaezelandiae* females, alleles were detected by microsatellite analysis that are characteristic for *A. crassus*, suggesting the hybrid origin of these eggs. In contrast, none of the eggs isolated from *A. crassus* females possessed alleles different from those found in *A. crassus* adults, but it was revealed that one female can be inseminated by several males.

**Conclusion:**

Our results show that *A. crassus* and *A. novaezelandiae* can co-infect a single eel and can mature together in the same swim bladder. We also provide evidence for the possibility of hybridization of *A. crassus* males with *A. novaezelandiae* females. Therefore, hybridization might be an explanation for the disappearance of *A. novaezelandiae* from Lake Bracciano.

## Findings

### Background

Nematodes of the genus *Anguillicola* are eel-specific swim bladder parasites using copepods as intermediate hosts (some *Anguillicola* spp. were moved to the genus *Anguillicoloides* according to [1], but recent molecular findings support the original taxonomy [2]). In eel species as final hosts, larval stages are found in the swim bladder wall while adult stages migrate into the swim bladder lumen and feed on the host’s blood [3]. The best studied and most abundant species is *Anguillicola crassus*[4], which was introduced to Europe in the early 1980s through the importation of living eels from Asia [5-8]. The original host of *A. crassus*, the Japanese eel (*Anguilla japonica*), is well adapted to the parasite and can limit parasite load, for example by massive encapsulation of larval stages [9,10]. In contrast, high infection intensities and prevalences were found in European eels (*Anguilla anguilla*), in which the nematode causes severe damage of the swim bladder wall, a general stress response and mortality of the host [5,11-13]. Therefore, the parasite is expected to threaten European eel populations, especially as the loss of swim bladder function might impair the spawning migration of eels [12,14].

*A. crassus* seems to be the most efficient invader of the genus, but it was not the first to be introduced to Europe. As early as 1975, Short-finned eels (*Anguilla australis*) from New Zealand infected with *Anguillicola novaezelandiae* were released into Lake Bracciano in Italy [15,16], where the parasite was able to infect European eels and built a stable population in the lake [17,18]. *A. novaezelandiae* showed high prevalence and intensity of infection in European eels, but there are no records of any damage to the swim bladder. To the best of our knowledge, there is no record of *A. novaezelandiae* being established in waters outside of Lake Bracciano, as this lake is not connected to other waterbodies. In 1993, *A. crassus* was described for the first time in Lake Bracciano, but no mixed infections of eels with both *Anguillicola* species were recorded [17].

Subsequently, *A. crassus* became the dominant species in the lake and so far *A. novaezelandiae* was not found any more [17,19]. One possible explanation is that *A. crassus* outcompeted and replaced *A. novaezelandiae*, but the two species might have also formed viable hybrids that resemble *A. crassus* morphologically. To assess the latter hypothesis we performed a laboratory infection experiment with subsequent microsatellite analysis, to test i) if *A. crassus* and *A. novaezelandiae* can co-infect the same eel and ii) if they are able to mate and produce hybrid offspring.

## Methods

### Infection experiment

European eels (*Anguilla anguilla*) were obtained from a commercial fish farm (Albe Fischfarm, Haren/Rütenbrock, Germany), where no cases of *Anguillicola crassus* infections were ever reported. To confirm the absence of parasites before the experiment, ten eels from the stock were killed, dissected and examined for the presence of parasites.

*Anguillicola novaezelandiae* collected from *Anguilla australis* originating from New Zealand and *A. crassus* collected from *A. anguilla* from lake Müggelsee in Berlin, Germany, were used for the laboratory life cycles. Infective third stage larvae (L3) of *A. crassus* and *A. novaezelandiae* were produced by the method of Haenen *et al*. [20].

For the experiment, one European eel (40 cm, 106 g) was infected with 10 L3 of *A. crassus* and 10 L3 of *A. novaezelandiae* by administering L3 with a stomach tube (1.5 mm diameter; B. Braun Melsungen AG) as described in [21]. The eel was kept in a 80 L fish tank at 20°C and was fed twice a week for a period of 222 days in order to guarantee the presence of second stage larvae for molecular analyses (for details on the life cycle see [22]). After 222 days post infection (dpi), the eel was killed by decapitation and examined immediately for infection of the swim bladder. Adult parasites were counted and sex was determined. For storage, worms were fixed separately in 70% ethanol. *Anguillicola* species were identified morphologically according to [1]. The identification was done without knowledge about the PCR results to avoid bias of the investigator. The infection experiment was conducted in compliance with national and institutional guidelines for the care and use of animals.

### Molecular species identification

Small pieces of the cuticle or the pharynx were cut off from each adult nematode recovered from the eel using sterile technique. Care was taken not to carry-over intestinal content of the worms. DNA was extracted from each sample using a JETQUICK DNA Clean-Up Spin Kit (Genomed) according to manufacturer’s instructions. Species-specific primers targeting *cox I* were designed for both nematode species according to a multiple alignment including *cox I* sequences from *A. crassus* (GenBank accession no.: EU376921), *Anguillicola globiceps* (JF805673), *Anguillicola papernai* (JF805697), *Anguillicola australiensis* (JF805640), *A. novaezelandiae* isolate from Tasmania (JF805629), *A. novaezelandiae* New Zealand isolate (JX868555) from the present study and European eel (HQ600683). Sequences of *A. crassus* specific primers were: crasscox for 5^′^-CCT TTT GTT AGG TGA TGG GCA A-3^′^, crasscox rev 5^′^-TAG CGA GAT CAA CAC TTA TAC CAG-3^′^, amplifying a product of 303 bp and for *A. novaezelandiae* (New Zealand isolate): novcox for 5^′^-ATT GGG TGA CGG CCA GTT ATA-3^′^, novcox rev 5^′^-ACT TAT ATG CTC CAG AGT AAT AGA ACT A-3^′^, amplifying a product of 404 bp. PCR conditions were optimized and specificity of primers was tested. One 20 μl PCR reaction mix contained 4 μl of 5x Crimson Taq buffer (New England Biolabs), 0.2 mM dNTP mix (New England Biolabs), 0.5 μM of each primer 0.5 U Crimson Taq (New England Biolabs) and 1 μl template DNA. The mix was topped up to 20 μl with PCR grade water. PCR was conducted on a TGradient thermocycler (Biometra) with the same program for both primer pairs: 95°C for 5 min, 35 cycles of 95°C, 58°C and 72°C each for 45s and a final elongation at 72°C for 5 min. PCR products were analysed by standard agarose gel electrophoresis.

### Microsatellite analysis

Microsatellite markers for *A. crassus* were previously developed by [23]. We tested those seven markers also for *A. novaezelandiae* and selected two markers (AcrCT04 and AcrCA102) that produced a clearly distinguishable pattern of PCR products for the two species. Each forward primer was labelled at the 5^′^-end with a fluorescent dye (FAM for AcrCT04 and HEX for AcrCA102).

To analyse the allelic pattern of the adult worms, their DNA was amplified with AcrCT04 and AcrCA102 according to [23]. For characterization of the offspring, ten eggs were dissected from each uterus of female nematodes. Eggs were washed by repeated transfers to drops of clean distilled water on a sterile petri dish. Subsequently, single eggs were placed in reaction tubes, DNA was extracted as described above and amplified with AcrCT04 and AcrCA102. PCR products were purified with a JETQUICK PCR Product Purification Spin Kit (Genomed). Products of AcrCT04 and AcrCA102 were pooled and sent for analysis on an Applied Biosystems DNA Analyzer ABI3730 (GATC). Fluorescent peaks were analysed with the Peak Scanner™ Software v1.0 (Applied Biosystems).

To check for contamination of egg DNA from one nematode species with DNA from the other, all eggs were tested with the *cox I*-PCR. As *cox I* is a mitochondrial marker, it should show the identity of the female irrespective of a possible hybrid origin.

## Results and discussion

### Infection experiment

Both primer pairs designed according to the *cox I* sequence of *A. crassus* and *A. novaezelandiae* were species specific and did not cross react with eel DNA.

In total, 13 adult nematodes were isolated from the swim bladder of the eel, five of which were female and eight male. One of the females was found dead and partly decomposed, therefore this specimen was not used for further analyses. No larval stages (L3 or L4) were detected in the swim bladder wall. The morphological species identification was in accordance with the *cox I* PCR results. Only the DNA from one female produced a strong band for *A. novaezelandiae* and an additional faint band with the crasscox-primers, indicating contamination of the sample. This problem remained even though new DNA was extracted and tested again twice from this specimen (Table 1). As the *A. crassus*-band was faint, we assumed that this specimen was *A. novaezelandiae*, in compliance with the result of the morphological identification. Two female and four male individuals were identified for each species, which equates to a recovery rate of 60% (ten third stage larvae from each species were used for infection) for each species with an overall recovery rate of 65%, considering also the dead female. This shows that co-infection of the final host with both species is possible, and might have also occurred in Lake Bracciano.

**Table 1:** Results of morphological and molecular examination of adult nematodes

**No.**	***A. crassus***						***A. novaezelandiae***						**dead ♀**
	**AC1**	**AC2**	**AC3**	**AC4**	**AC5**	**AC6**	**AN1**	**AN2**	**AN3**	**AN4**	**AN5**	**AN6**	**--**
Sex	♀	♀	♂	♂	♂	♂	♀	♀	♂	♂	♂	♂	♀
Morphology	AC	AC	AC	AC	AC	AC	AN	AN	AN	AN	AN	AN	ND
*cox I* PCR	AC	AC	AC	AC	AC	AC	AN	AN	AN	AN	AN	AN	ND
									AC*				
Alleles AcrCT04	123	135	123	135	135	135	109	109	109	109	109	109	ND
	198	163	146	159	--	198	--	--	--	--	--	--	
Alleles AcrCA102	325	305	305	305	305	305	--	--	--	--	--	--	ND
	--	325	325	--	325	325	--	--	--	--	--	--	

AC: *A. crassus*, AN: *A. novaezelandiae*; numbers indicate allele size in bp; ND: not determined; *faint band.

### Microsatellite analysis

According to the analysis of adult nematodes, allele sizes for marker AcrCT04 ranged from 123 bp – 198 bp for *A. crassus*, while a single 109 bp allele was characteristic for *A. novaezelandiae* specimens. AcrCA102 amplified two alleles, one 305 bp and one 325 bp. These were detected only in *A. crassus* samples (Table 1). Analysis of microsatellite patterns of the eggs revealed information on parental combinations. Only “*A. crassus-*alleles” were found in the eggs analysed from the females AC1 and AC2, showing that species boundaries were not crossed. Characteristic alleles of *A. crassus* males, allowed the identification of the potential father in some cases (Table 2). Apparently, one female was inseminated by several males, giving rise to eggs fertilized by different sperm (e.g. AC3 and AC6 for *A. crassus* female 1; see also Table 2).

**Table 2:** Results of microsatellite analysis for eggs

**AC ♀ no. 1**	**egg 1**	**egg 2**	**egg 3**	**egg 4**	**egg 5**	**egg 6**	**egg 7**	**egg 8**	**egg 9**	**egg 10**
*cox I* PCR	AC	AC	AC	AC	AC	AC	AC	AC	AC	AC
Alleles AcrCT04	123	146	123	ND	123	123	123	135	198	123
	135	198	198		198	198	198	198	--	135
Alleles AcrCA102	325	305	305	ND	325	305	305	325	305	305
	--	325	325		--	325	325	--	325	325
potential fathers	AC4, AC5, AC6	AC3	AC3, AC6		AC3, AC6	AC3, AC6	AC3, AC6	AC4, AC5, AC6	AC6	AC4, AC5, AC6
**AC ♀ no. 2**	**egg 1**	**egg 2**	**egg 3**	**egg 4**	**egg 5**	**egg 6**	**egg 7**	**egg 8**	**egg 9**	**egg 10**
*cox I* PCR	AC	AC	AC	AC	AC/**AN**	AC	AC	AC	AC/**AN**	AC
Alleles AcrCT04	163	135	C	146	123	135	C	135	159	ND
	198	--		163	163	146		159	163	
Alleles AcrCA102	325	305		325	305	325		305	305	ND
	--	325		--	325	--		325	--	
potential fathers	AC6	AC4, AC5, AC6		AC3	AC3	AC3		AC4	AC4	
**AN ♀ no. 1**	**egg 1**	**egg 2**	**egg 3**	**egg 4**	**egg 5**	**egg 6**	**egg 7**	**egg 8**	**egg 9**	**egg 10**
*cox I* PCR	AN	AN	AN	AN	AN	AN	AN	AN	AN/**AC**	AN
Alleles AcrCT04	109	109	109	109	109	109	109	109	ND	109
	**123**	--	**146**	**123**	--	--	**135**	--		**146**
Alleles AcrCA102	**325**	--	**325**	**325**	--	--	**305**	--	ND	**305**
	--		--	--			--			--
potential fathers	AC3		AC3	AC3			AC4, AC5, AC6			AC3
**AN ♀ no. 2**	**egg 1**	**egg 2**	**egg 3**	**egg 4**	**egg 5**	**egg 6**	**egg 7**	**egg 8**	**egg 9**	**egg 10**
*cox I* PCR	AN	AN	AN	AN	AN	AN	AN/**AC**	AN	AN	AN/**AC**
Alleles AcrCT04	109	109	109	109	109	109	ND	109	109	ND
	--	--	**198**	**159**	--	**198**		**198**	--	
Alleles AcrCA102	--	--	**305**	**305**	--	**325**	ND	**305**	--	ND
			--	--		--		--		
potential fathers			AC6	AC4		AC6		AC6		

AC: *A. crassus*, AN: *A. novaezelandiae*; numbers indicate allele size in bp; C: more than 2 alleles were present, indicating contamination with DNA of 2^nd^ individual; alleles indicating hybrids or PCR results showing contaminations are highlighted in bold.

In the offspring of the two *A. novaezelandiae* females AN1 and AN2, alleles characteristic for *A. crassus* were detected. In five out of nine eggs from AN1 and in four out of eight eggs from AN2, alleles different from the 109 bp allele were amplified by AcrCT04. In all these individuals, either the 305 bp or the 325 bp “*A. crassus*-allele” amplified by AcrCA102 was found as well, strongly indicating hybrid origin of these eggs, attributable to at least three different *A. crassus* males. Contamination was excluded in these cases by *cox I*–PCR that detected only *A. novaezelandiae* (maternal) mitochondrial DNA in the hybrids (Table 2).

In some cases, no PCR product was obtained from single eggs (Table 2). Most likely, DNA-extraction was unsuccessful, because eggs were lost during collection and washing as they easily stick in pipette tips and other plastics. For egg no. 3 and egg no. 7 of *A. crassus* female no. 2, more than two alleles were detected by marker AcrCT04, which revealed contamination of the sample with DNA of another *A. crassus* individual. The *cox I* PCR also indicated a weak *A. novaezelandiae* contamination (faint bands) in DNA from eggs no. 5 and no. 9 isolated from AC female no. 2, although no AN alleles were detected in these samples (Table 2). Also, contamination of some DNA samples from *A. novaezelandiae* eggs with *A. crassus* DNA was detected by *cox I*-PCR (AN1: egg no. 9, AN2: egg no. 7 and no. 10; see Table 2). These eggs were not taken into account for evaluation of the microsatellite data. Reasons for the contaminations found in one adult nematode (AN3) and several eggs might have been carry over of tissue or eggs from other individuals during sampling or DNA extraction, remainders of sperm in the uteri, or errors during PCR setup. Nevertheless, most individuals were contamination free and allowed reliable interpretation of the results.

These results provide evidence that *A. crassus* and *A. novaezelandiae* can form hybrids, at least in laboratory infections. As *A. crassus* originated from Asia and *A. novaezelandiae* from New Zealand, these species would not have met under natural conditions without human influence. Therefore, evolving a morphological or behavioural reproductive barrier between the two species was not necessary. It is not clear, whether there is some pre- or postzygotic barrier for *A. novaezelandiae* males to fertilize *A. crassus* females, or if the lack of hybrids from *A. crassus* females was a coincidental result of our study. Further experimental double infections of eels with both *Anguillicola* spp. will help to answer this question.

Hybridization within different groups of helminth parasites is a well-known phenomenon. In laboratory experiments, hybridization was proven for different *Schistosoma* spp. [24-27] and two other trematodes, *Fasciola hepatica* and *Fasciola gigantica*[28]. In most of these laboratory studies, hybrids showed reduced survival and impaired fertility already in the F1 or F2 generation. However, hybridization among parasites also occurs under natural conditions and was observed in monogeneans [29], schistosomes [30] and nematodes (*Anisakis*) [31]. The eggs isolated from the females from the present study were fixed in ethanol and therefore it could not be tested, if the hybrid larvae produced are viable, infective, and if they develop into fertile adults, but these issues will be investigated in further studies. Therefore, it can only be speculated if hybridization between *A. crassus* and *A. novaezelandiae* is an explanation for what happened in Lake Bracciano.

A case of replacement of one species by another through introgressive hybridization among helminth parasites was described for *Schistosoma haematobium* and *Schistosoma intercalatum* in a part of Cameroon where *S. intercalatum* was completely replaced by the introduced *S. haematobium* and the hybrids of both species within about 30 years [32,33]. Laboratory experiments proved that hybrids of *S. haematobium* and *S. intercalatum* were more successful in mating competition than both *S. intercalatum* and, to a lesser extent, *S. haematobium*[34]. A similar scenario might be possible for Lake Bracciano, though the replacement of *A. novaezelandiae* by *A. crassus* occurred much faster. But incompatibility of *A. novaezelandiae* males with *A. crassus* females might quickly lead to a dominance of *A. crassus* genes in the population. The hybrid offspring would also be a selective disadvantage for *A. novaezelandiae* females, even if these hybrids are not viable. Of course alternative explanations for the disappearance of *A. novaezelandiae* from Lake Bracciano are likely such as a faster development of larval stages in the copepod intermediate hosts or a broader spectrum of suitable intermediate hosts for *A. crassus*. These and other life-cycle parameters have to be studied in detail before introgressive hybridization can be figured out as the main reason for the disappearance of *A. novaezelandiae*.

## Conclusions

Results of the present study show that *A. crassus* and *A. novaezelandiae* might occur in the same host in areas of sympatry like Lake Bracciano. According to our results, hybridization is possible between *A. novaezelandiae* females and *A. crassus* males, but not vice versa. This finding would fit well to the scenario of Lake Bracciano, where *A. novaezelandiae* seemed to have disappeared. Further laboratory hybridization-experiments are planned to test if this hybrid offspring is viable and fertile, and to analyse their morphology if adult nematodes develop.

## Competing interests

The authors declare that they have no competing interests.

## Authors’ contributions

DG was involved in dissecting the nematodes and egg sampling, primer design, molecular work, microsatellite analysis and writing the manuscript. KD and BS established and maintained the laboratory life-cycle of nematodes and contributed to writing by corrections and critical comments. KD conducted the infection experiments, eel and nematode dissection and sampling, as well as morphological identification of nematodes and sequencing of *cox I*. BS had a substantial role in conception of the study and guidance of the practical work. All authors read and approved the manuscript.

## References

[B1] Moravec F Dracunculoid and Anguillicoloid Nematodes Parasitic in Vertebrates 2006 Prague: Academia

[B2] Laetsch DR Heitlinger EG Taraschewski H Nadler SA Blaxter ML The phylogenetics of Anguillicolidae (Nematoda: Anguillicolidea), swimbladder parasites of eels BMC Evol Biol 2012 12 60 10.1186/1471-2148-12-60 22559142 PMC3503875

[B3] De Charleroy D Grisez L Thomas K Belpaire C Ollevier FP The life cycle of *Anguillicola crassus* Dis Aquat Organ 1990 8 77 84

[B4] Kuwahara A Itagaki H Itagaki H Studies of a nematode parasitic in the air bladder of the eel: 1. Description of Anguillicola crassus n. sp. (Philometridae, Anguillicolidae) Jpn J Parasitol 1974 23 275 279

[B5] Kennedy CR The pathogenic helminth parasites of eels J Fish Dis 2007 30 319 334 10.1111/j.1365-2761.2007.00821.x 17498176

[B6] Kirk RS The impact of *Anguillicola crassus* on European eels Fish Manage Ecol 2003 10 385 394 10.1111/j.1365-2400.2003.00355.x

[B7] Koops H Hartmann F *Anguillicola*-infestations in Germany and in German eel imports J Appl Ichthyol 1989 5 41 45 10.1111/j.1439-0426.1989.tb00568.x

[B8] Taraschewski H Moravec F Lamah T Anders K Distribution and morphology of two helminths recently introduced into European eel populations: *Anguillicola crassus* (Nematoda, Dracunculoidea) and *Paratenuisentis ambiguus* (Acanthocephala, Tenuisentidae) Dis Aquat Organ 1987 3 167 176

[B9] Knopf K The swimbladder nematode *Anguillicola crassus* in the European eel *Anguilla anguilla* and the Japanese eel *Anguilla japonica*: differences in susceptibility and immunity between a recently colonized host and the original host J Helminthol 2006 80 129 136 10.1079/JOH2006353 16768856

[B10] Heitlinger EG Laetsch DR Weclawski U Han YS Taraschewski H Massive encapsulation of larval *Anguillicoloides crassus* in the intestinal wall of Japanese eels Parasit Vectors 2009 2 48 10.1186/1756-3305-2-48 19832983 PMC2766375

[B11] Egusa S Notes on the culture of European eel (*Anguilla anguilla*) in Japanese eel farming ponds J Conseil Int Explor Mer 1979 174 51 58

[B12] Sures B Knopf K Parasites as a threat to freshwater eels? Science 2004 304 208 209 10.1126/science.304.5668.209 15073357

[B13] Sures B Knopf K Kloas W Induction of stress by the swimbladder nematode *Anguillicola crassus* in European eels, *Anguilla anguilla*, after repeated experimental infection Parasitology 2001 123 179 184 11510683 10.1017/s003118200100823x

[B14] Palstra AP Heppener DFM van Ginneken VJT Székely C van Den Thillart GEEJ Swimming performance of silver eels is severely impaired by the swim-bladder parasite *Anguillicola crassus* J Ex Mar Biol Ecol 2007 352 244 256 10.1016/j.jembe.2007.08.003

[B15] Moravec F Taraschewski H Revision of the genus *Anguillicola* Yamaguti, 1935 (Nematoda: Anguillicolidae) of the swimbladder of eels, including descriptions of two new species, A. novaezelandiae sp. n. and A. papernai sp. n. Folia Parasitol 1988 35 125 146 3169643

[B16] Welcomme RL Register of international transfers of inland fish species FAO Fish Tech Pap 1981 213 120

[B17] Moravec F Di Cave D Orecchia P Paggi L Present occurrence of *Anguillicola novaezelandiae* (Nematoda, Dracunculoidea) in Europe and its development in the intermediate host Folia Parasitol 1994 41 203 208 7883252

[B18] Paggi L Orecchia P Minervini R Mattiucci S Appearance of *Anguillicola australiensis* Johnston and Mawson, 1940 (Dracuncoloidea: Anguillicolidae) in *Anguilla anguilla* of Lake Bracciano Parassitologia 1982 24 139 144 6926929

[B19] Marcel Münderle Ökologische, morphometrische und genetische Untersuchungen an Populationen des invasiven Schwimmblasen-Nematoden Anguillicola crassus aus Europa und Taiwan. PhD Thesis 2005 University of Karlsruhe, Faculty for Chemistry and Bioscience

[B20] Haenen OLM Van Wijngaarden TAM Borgsteede FHM An improved method for the production of infective third-stage juveniles of *Anguillicola crassus* Aquaculture 1994 123 163 165 10.1016/0044-8486(94)90128-7

[B21] Sures B Knopf K Individual and combined effects of Cd and 3,3′,4,4′,5-pentachlorobiphenyl (PCB 126) on the humoral immune response in European eel (*Anguilla anguilla*) experimentally infected with larvae of *Anguillicola crassus* (Nematoda) Parasitology 2004 128 445 454 10.1017/S003118200300475X 15151150

[B22] Knopf K Würtz J Sures B Taraschewski H Impact of low water temperature on the development of *Anguillicola crassus* in the final host *Anguilla anguilla* Dis Aquat Organ 1998 33 143 149 9722403 10.3354/dao033143

[B23] Wielgoss S Sanetra M Meyer A Wirth T Isolation and characterization of short tandem repeats in an invasive swimbladder nematode, parasitic in Atlantic freshwater eels, *Anguillicola crassus* Mol Ecol Notes 2007 7 1051 1053 10.1111/j.1471-8286.2007.01773.x

[B24] Fan PC Lin LH Hybridization of *Schistosoma mansoni* and *Schistosoma japonicum* in mice Southeast Asian J Trop Med Public Health 2005 36 89 96 15906648

[B25] Leroux PL Hybridization of *Schistosoma mansoni* and *S. rodhaini* Trans Roy Soc Trop Med Hyg 1954 48 3 4

[B26] Taylor MG Hybridization experiments on five species of African schistosomes J Helminthol 1970 44 253 314 5505355 10.1017/s0022149x00021969

[B27] Théron A Hybrids between *Schistosoma mansoni* and *Schistosoma rodhaini*: characterization by cercarial emergence rhythms Parasitology 1989 99 225 228 10.1017/S0031182000058674 2594413

[B28] Itagaki T Ichinomiya M Fukuda K Fusyuku S Carmona C Hybridization experiments indicate incomplete reproductive isolating mechanism between *Fasciola hepatica* and *Fasciola gigantica* Parasitology 2011 138 1278 1284 10.1017/S0031182011000965 21767436

[B29] Barson M Prikrylová I Vanhove MPM Huyse T Parasite hybridization in African Macrogyrodactylus spp. (Monogenea, Platyhelminthes) signals historical host distribution Parasitology 2010 137 1585 1595 10.1017/S0031182010000302 20444301

[B30] Morgan JAT DeJong RJ Lwambo NJS Mungai BN Mkoji GM Loker ES First report of a natural hybrid between *Schistosoma mansoni* and *S. rodhaini* J Parasitol 2003 89 416 418 10.1645/0022-3395(2003)089[0416:FROANH]2.0.CO;2 12760671

[B31] Martín-Sánchez J Artacho-Reinoso ME Díaz-Gavilán M Valero-López A Structure of *Anisakis simplex* s.l. populations in a region sympatric for *A. pegreffii* and *A. simplex* s.s. Absence of reproductive isolation between both species Mol Biochem Parasitol 2005 141 155 162 10.1016/j.molbiopara.2005.02.005 15850698

[B32] Southgate VR Rollinson D Interactions of *Schistosoma haematobium* and *S. intercalatum* in Loum, Cameroon Proceedings of the Third European Multicolloquium of Parasitology: 7-13 September 1980 1981 Cambridge: Cambridge University Press 24

[B33] Tchuem Tchuenté LA Southgate VR Njiokou F Njiné T Kouemeni LE Jourdane J The evolution of schistosomiasis at Loum, Cameroon: replacement of *Schistosoma intercalatum* by *S. haematobium* through introgressive hybridization Trans Roy Soc Trop Med Hyg 1997 91 664 665 10.1016/S0035-9203(97)90513-7 9509173

[B34] Webster BL Southgate VR Mating interactions of *Schistosoma haematobium* and *S. intercalatum* with their hybrid offspring Parasitology 2003 126 327 338 10.1017/S0031182002002883 12741512

